# Baicalein inhibits fibronectin-induced epithelial–mesenchymal transition by decreasing activation and upregulation of calpain-2

**DOI:** 10.1038/s41419-019-1572-7

**Published:** 2019-04-18

**Authors:** Yan Chen, Lin Chen, Duanyang Hong, Zongyue Chen, Jingyu Zhang, Lingyun Fu, Di Pan, Yanyan Zhang, Yini Xu, Shiquan Gan, Chaoda Xiao, Ling Tao, Xiangchun Shen

**Affiliations:** 1 The Department of Pharmacology of Materia Medica (the State Key Laboratory of Functions and Applications of Medicinal Plants, the High Educational Key Laboratory of Guizhou Province for Natural Medicinal Pharmacology and Druggability), School of Pharmaceutical Sciences, Guizhou Medical University, University Town, Guian New District, Guizhou China; 2 The High Efficacy Application of Natural Medicinal Resources Engineering Center of Guizhou Province, School of Pharmaceutical Sciences, Guizhou Medical University, University Town, Guian New District, Guizhou China; 3 The Union Key Laboratory of Guiyang City-Guizhou Medical University, School of Pharmaceutical Sciences, Guizhou Medical University, University Town, Guian New District, Guizhou China; 4 The Key Laboratory of Optimal Utilization of Natural Medicine Resources, School of Pharmaceutical Sciences, Guizhou Medical University, University Town, Guian New District, Guizhou China

**Keywords:** Pharmacodynamics, Drug development

## Abstract

The extracellular matrix protein fibronectin (FN) facilitates tumorigenesis and the development of breast cancer. Inhibition of the FN-induced cellular response is a potential strategy for breast cancer treatment. In the present study, we investigated the effects of the flavonoid baicalein on FN-induced epithelial–mesenchymal transition (EMT) in MCF-10A breast epithelial cells and in a transgenic mouse MMTV-polyoma middle T antigen breast cancer model (MMTV-PyMT). Baicalein inhibited FN-induced migration, invasion, and F-actin remodeling. Baicalein also suppressed FN-induced downregulation of the epithelial markers E-cadherin and ZO-1 and upregulation of the mesenchymal markers N-cadherin, vimentin, and Snail. Further investigation revealed that calpain-2 was involved in baicalein suppression of FN-induced EMT. Baicalein significantly decreased FN-enhanced calpain-2 expression and activation by suppressing its plasma membrane localization, substrate cleavage, and degradation of its endogenous inhibitor calpastatin. Overexpression of calpain-2 in MCF-10A cells by gene transfection partially blocked the inhibitory effect of baicalein on FN-induced EMT changes. In addition, baicalein inhibited calpain-2 by decreasing FN-increased intracellular calcium ion levels and extracellular signal-regulated protein kinases activation. Baicalein significantly decreased tumor onset, growth, and pulmonary metastasis in a spontaneous breast cancer MMTV-PyMT mouse model. Baicalein also reduced the expression of FN, calpain-2, and vimentin, but increased E-cadherin expression in MMTV-PyMT mouse tumors. Overall, these results revealed that baicalein markedly inhibited FN-induced EMT by inhibiting calpain-2, thus providing novel insights into the pharmacological action and mechanism of baicalein. Baicalein may therefore possess therapeutic potential for the treatment of breast cancer though interfering with extracellular matrix–cancer cell interactions.

## Introduction

Breast cancer is the most common cancer diagnosis in women worldwide^1^. The main adjuvant therapies after surgical treatment for breast cancer involve chemotherapy, hormonal therapy, and molecular targeted therapy, which depends on the patient’s hormone receptor or human epidermal growth factor (EGF) receptor 2 status^2^. Although breast cancer mortality rates have declined in line with improvements in early detection and treatment, not all patients have benefited from these improvements, and it remains the second leading cause of cancer-related death among women in the United States^3^. New strategies for breast cancer therapy are therefore urgently required.

Tumor cells constantly interact with the extracellular matrix (ECM) in the tumor microenvironment, which mediates numerous processes to promote tumor progression^4^. Fibronectin (FN) is a key component of the tumor ECM facilitating tumor formation, proliferation, angiogenesis, and metastasis, especially in breast cancer^5^, and FN mRNA and protein levels have been shown to be increased in breast tumor stroma, but are not expressed in normal adult breast tissue^6^. FN expression in primary breast tumors was also positively correlated with an invasive and metastatic phenotype^7^ and negatively associated with survival and clinical outcome in breast cancer patients^8,9^. FN transmits ECM signals to stimulate mammary epithelial cells to lose their growth-arrested polarized characteristic, acquire tumor-like behavior, proliferate excessively, and eventually form a disturbed acinar structure in three-dimensional culture^10^, whereas anti-FN antibodies induce T4-2 tumorigenic cells to form an organized polarized acinar identical to normal breast epithelial cells^11^. Therapeutic immunization using an antibody against extra domain-A of FN attenuated tumor burden and lung metastases in mouse mammary tumor virus polyoma virus middle T (MMTV-PyMT) mice^12^. These observations suggest that FN is a critical stimulator for breast tumor initiation and development.

Epithelial–mesenchymal transition (EMT) involves the trans-differentiation of stationary polarized epithelial cells into disorganized motile mesenchymal cells, via the loss of tight cell–cell junctions and the acquisition of mobile and invasive abilities^13^. EMT is therefore envisioned as a differentiation or morphogenetic process in embryogenesis, tissue repair, and remodeling, specifically contributing to tumor progression^14^. Most breast cancers are carcinomas of epithelial origin, in which the progression to malignancy appears to exploit a pathological EMT process^15^. FN is an important source of EMT-regulatory cues in cancer cells via specific structural modifications or alterations, or via crosslinking with collagen to mediate mechanotransduction^16^. FN alone or the assembly of FN fibrils, which contain the growth factor-binding domain and serve to localize transforming growth factor-β1 (TGF-β1) signaling, induces EMT in human mammary epithelial cells^17,18^. These studies therefore imply that suppression of FN-induced EMT is a promising approach for inhibiting breast cancer progression.

Calpains comprise a conserved family of calcium‑dependent neutral cysteine proteases with limited proteolytic activity, including two major canonical members, calpain-1 and calpain-2^19^. They promote various crucial processes for cancer development including cell survival, malignant transformation, metastasis, and angiogenesis^20^. Notably, they are also implicated in cancer cell EMT. Calpain proteases have been shown to induce proteolysis of the Rac activator Tiam1, leading to disassembly of adherens junctions in Src-mediated EMT^21^. Calpain-1 augments TGF-β1-induced EMT in human lung epithelial A549 cells through the phosphoinositide 3-kinase/Akt signaling pathway^22^. Calpain-2 regulates cortical actin remodeling to facilitate signal-regulated protein kinase (ERK)-induced phenotypic conversion of epithelial MDCK cells, from apical–basal polarity to autonomously migrating cells^23^. Calpain also participates in FN signal transduction. Calpain-2 has been reported to be a main transductor in FN-mediated focal adhesion kinase (FAK)/ERK1/2 signaling in A549 lung cancer cells^24^. We previously found that calpain inhibitors reversed FN‑induced EMT in breast cancer cells^25^. These studies indicate that decreasing calpain activity or expression may interfere with the FN-induced EMT response.

Baicalein is an active extract of the root of the traditional Oriental medicine *Scutellaria baicalensis* Georgi, which possesses effective anticancer properties^26^. Baicalein has shown potent effects on breast cancer by targeting multiple pathways, including inhibiting cell proliferation and metastasis, inducing cell cycle arrest and apoptosis, and preventing tumorigenesis and angiogenesis^26^. However, the effect of baicalein on ECM components such as FN-induced EMT in breast epithelial cells remains unknown. In this study, we investigated the ability of baicalein to suppress FN-induced EMT in MCF-10A cells and examined its effects on breast tumor initiation, growth, lung metastasis, and EMT changes in MMTV-PyMT mice with spontaneous mammary carcinomas. We also estimated the role of calpain-2 in the anti-EMT effect of baicalein, and its upstream ERK and Ca^2+^ events. The results suggest that baicalein may be a promising anti-breast cancer candidate with the potential to interfere with tumor cell ECM–cancer cell interactions.

## Results

### Baicalein inhibits FN-induced migration, invasion, F-actin remodeling, and EMT-related biomarker expression changes in MCF-10A cells

**Fig. 1:**
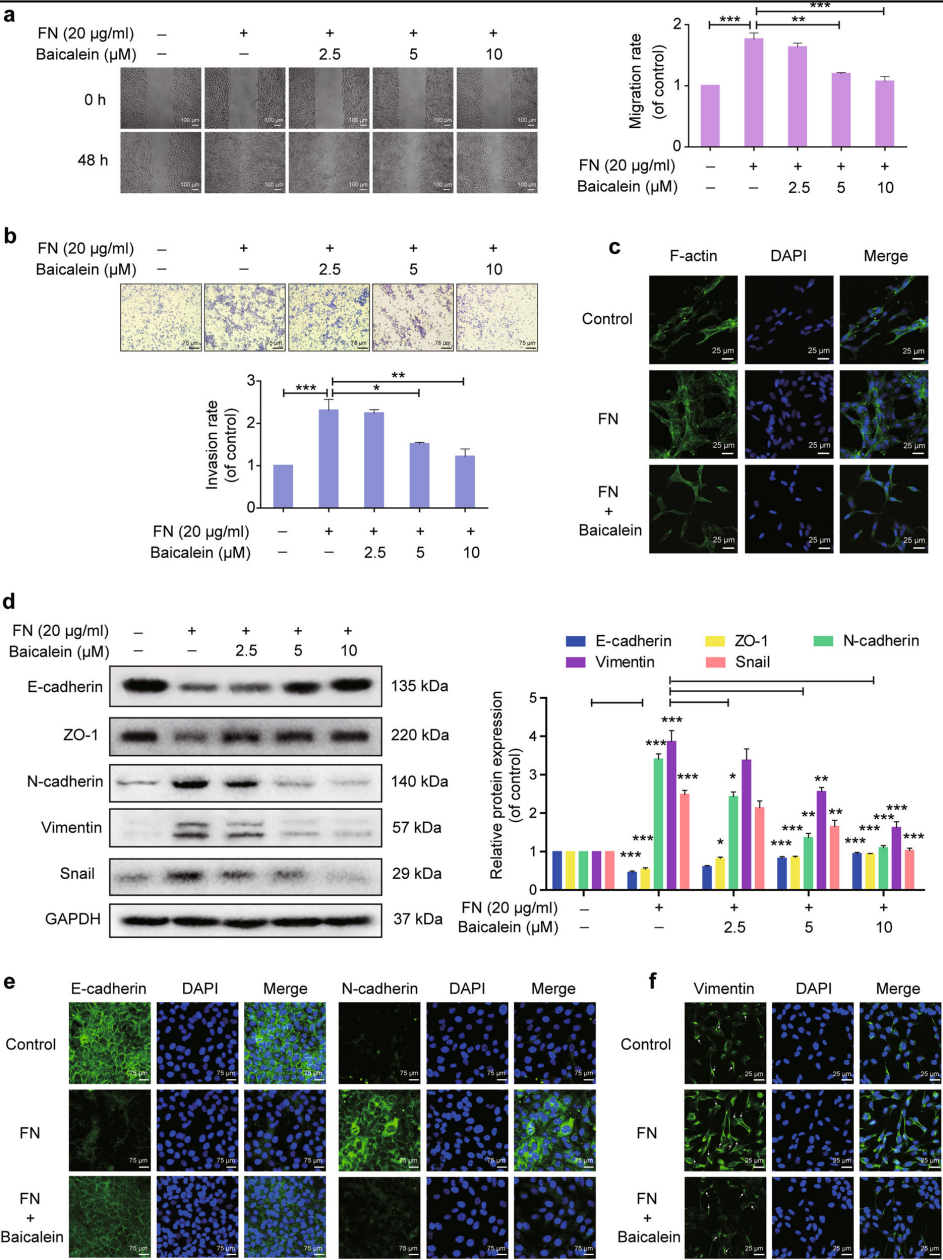
Effects of baicalein on fibronectin (FN)-induced epithelial–mesenchymal transition (EMT) in breast epithelial cells. Cells were plated on 20 μg ml^−1^ FN and treated with or without baicalein for 48 h. **a** Cell migration was measured using a wound-healing assay. Images were captured at 0 and 48 h after wounding (magnification, ×100, scale bars: 100 μm). **b** Cell invasion was investigated using the Matrigel-coated transwell model (magnification, ×200, scale bars: 75 μm). **c** Representative images from confocal microscopy analysis of F-actin organization. F-actin stained with FITC–phalloidin (green fluorescence) and cell nuclei stained with DAPI (blue fluorescence) were detected by confocal microscopy (TCS SP5; Leica, Mannheim, Germany) with Leica Application Suite Advanced Fluorescence acquisition software (original magnification ×800, scale bars: 25 μm). **d** Immunoblots showing expression of E-cadherin, ZO-1, N-cadherin, vimentin, and Snail in MCF-10A cells. Data represent densitometric quantification of EMT-related protein normalized with GAPDH and shown as the fold-change compared with control cells. **e** Representative immunofluorescence microscopy images of E-cadherin and N-cadherin after FN treatment with or without 10 μM baicalein for 48 h. Magnification, ×200, scale bars: 75 μm. **f** Representative confocal microscopy images of vimentin in cells after FN treatment with or without 10 μM baicalein for 48 h. Original magnification ×800, scale bars: 25 μm. Arrows indicate the network of vimentin filaments. Data are shown as mean ± SEM for three separate experiments. *P < 0.05, **P < 0.01, ***P < 0.001

As breast epithelial cell migration and invasion increase with increasing mesenchymal characteristics during breast cancer progression^27^, we tested the effects of baicalein on FN-enhanced cell migration and invasion. The concentrations of FN and baicalein used had no significant effect on the viability of MCF-10A cells (Supplementary Fig. S1). Baicalein inhibited FN-stimulated MCF-10A cell migration into the wounded space (Fig. 1a) and reduced FN-promoted MCF-10A cell invasion across a Matrigel-coated transwell membrane (Fig. 1b). The expression, localization, and activity of many cytoskeletal proteins are altered during EMT, leading to profound cytoskeleton rearrangements, which are required to increase cell motility and invasiveness^28^. F-actin was stained with Phalloidin-iFluor 488 and observed under confocal microscopy. After FN treatment, F-actin filaments appeared scattered throughout the cytoplasm, displaying a representative unstable cytoskeleton structure, but FN-induced changes in F-actin remodeling were reversed by baicalein treatment (Fig. 1c).

We further quantified the effects of baicalein on FN-induced EMT by examining the expression of several key epithelial and mesenchymal markers. FN treatment significantly reduced protein levels of the epithelial markers E-cadherin and ZO-1 and increased protein levels of the mesenchymal markers N-cadherin, vimentin, and Snail compared with the control group (Fig. 1d). Furthermore, FN, but not other tested ECM protein substrates such as collagen IV or laminin, induced E-cadherin upregulation and vimentin downregulation (Supplementary Fig. S2). However, co-treatment with baicalein reversed the FN-induced changes in EMT-related biomarkers. These influences of baicalein were further confirmed by immunofluorescence analysis. E-cadherin staining in the control cells indicated high expression and tight cell–cell junctions, which were decreased and partially disrupted by FN but restored by baicalein treatment (Fig. 1e). Similarly, FN-induced N-cadherin upregulation was also inhibited by baicalein (Fig. 1e). In addition, baicalein inhibited the FN-promoted increase in vimentin fluorescence intensity and reversed the reorganization of vimentin (Fig. 1f). Vimentin was organized into a complex and widespread network of filaments elongating to the periplasm following FN treatment, whereas vimentin filaments were scattered throughout the cytoplasm and only partially organized in baicalein-treated cells.

### Baicalein inhibits FN-promoted calpain-2 expression and activity

**Fig. 2:**
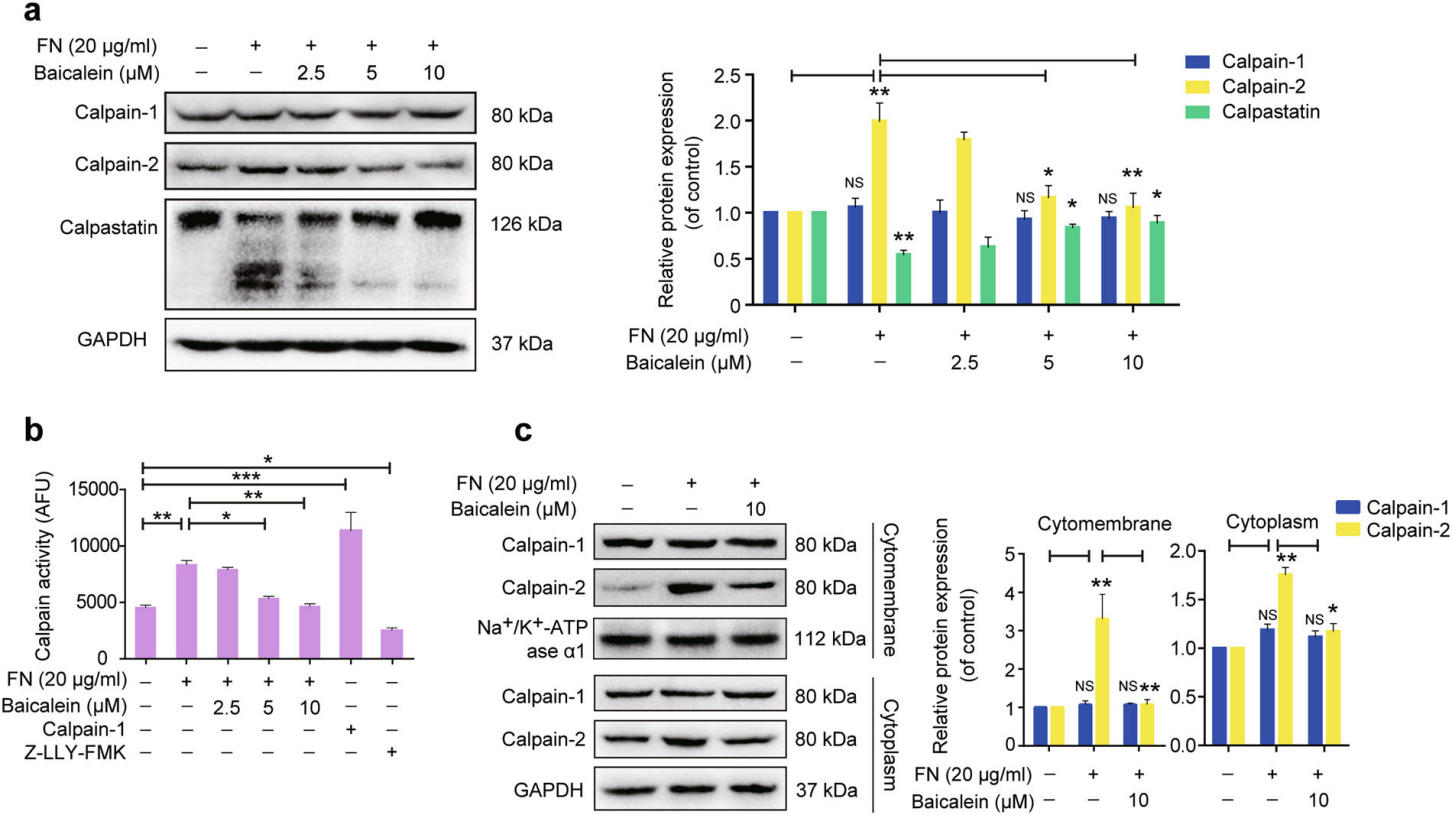
Effects of baicalein on fibronectin (FN)-enhanced calpain-2 protein expression and activity. **a** MCF-10A cells were treated with FN (20 μg ml^−1^) in the presence or absence of baicalein at the indicated concentrations for 48 h. The results were normalized to GAPDH expression and shown as the fold-change compared with control cells. **b** Calpain activity, in terms of arbitrary fluorescence units (AFUs), was measured by monitoring fluorescence assay (Ex/Em = 400/505 nm). Fluorescence was detected after FN (20 μg ml^−1^) treatment with or without baicalein at the indicated concentrations for 48 h. Calpain-1 and the calpain inhibitor Z-LLY-FMK were used as positive and negative controls, respectively. **c** Localization of calpain-1 and calpain-2 in the cytomembrane and cytoplasm were analyzed by western blotting and expression levels were normalized to Na^+^/K^+^-ATPase ɑ1 and GAPDH, respectively. The results are shown as fold-change compared with control cells. Data are shown as mean ± SEM for three separate experiments. *P < 0.05, **P < 0.01, ***P < 0.001

Calpain-2 has demonstrated a critical role in FN-induced signaling transduction and cellular responses^24,25^. We determined if FN regulates the calpain–calpastatin proteolytic system in the EMT response by examining protein expression levels of calpain-1, calpain-2, and their specific endogenous inhibitor calpastatin in MCF-10A cells. The results showed that calpain-2 expression increased after FN treatment for 24–48 h (Fig. 2a and Supplementary Fig. S3a); however, calpain-1 expression was not visibly altered. Calpastatin negatively regulates calpain activity, and can be proteolytically cleaved by activated calpains, thus preventing its inhibitory action^29^. Abundance of the 126 kDa full-length calpastatin protein reduced in parallel with the formation of a 50–60 kDa cleaved fragment following FN treatment for 24–48 h. (Fig. 2a and Supplementary Fig. S3a), indicating increased calpain proteolytic activity. We then examined intracellular calpain activity using the calpain substrate Ac-LLY-AFC, which releases free AFC upon cleavage by calpain and can be assessed by fluorescence detection. Treatment with FN for 24–48 h induced calpain activation in MCF-10A cells (Fig. 2b and Supplementary Fig. S3b). However, calpain-2 upregulation in coordination with calpastatin degradation was suppressed by co-treatment with baicalein (Fig. 2a). Baicalein also suppressed FN-promoted calpain activity (Fig. 2b). Calpain-2 activity is subject to post-translational control and partially accumulates on the plasma membrane in activating conditions^30,31^. FN stimulation increased calpain-2 expression in the cytomembrane compartments, and this localization was significantly decreased by baicalein (Fig. 2c). Baicalein also interfered with FN-induced upregulation of calpain-2 in the cytoplasm.

### Baicalein retards FN-induced increase in intracellular Ca^2+^ abundance and ERK activation

**Fig. 3:**
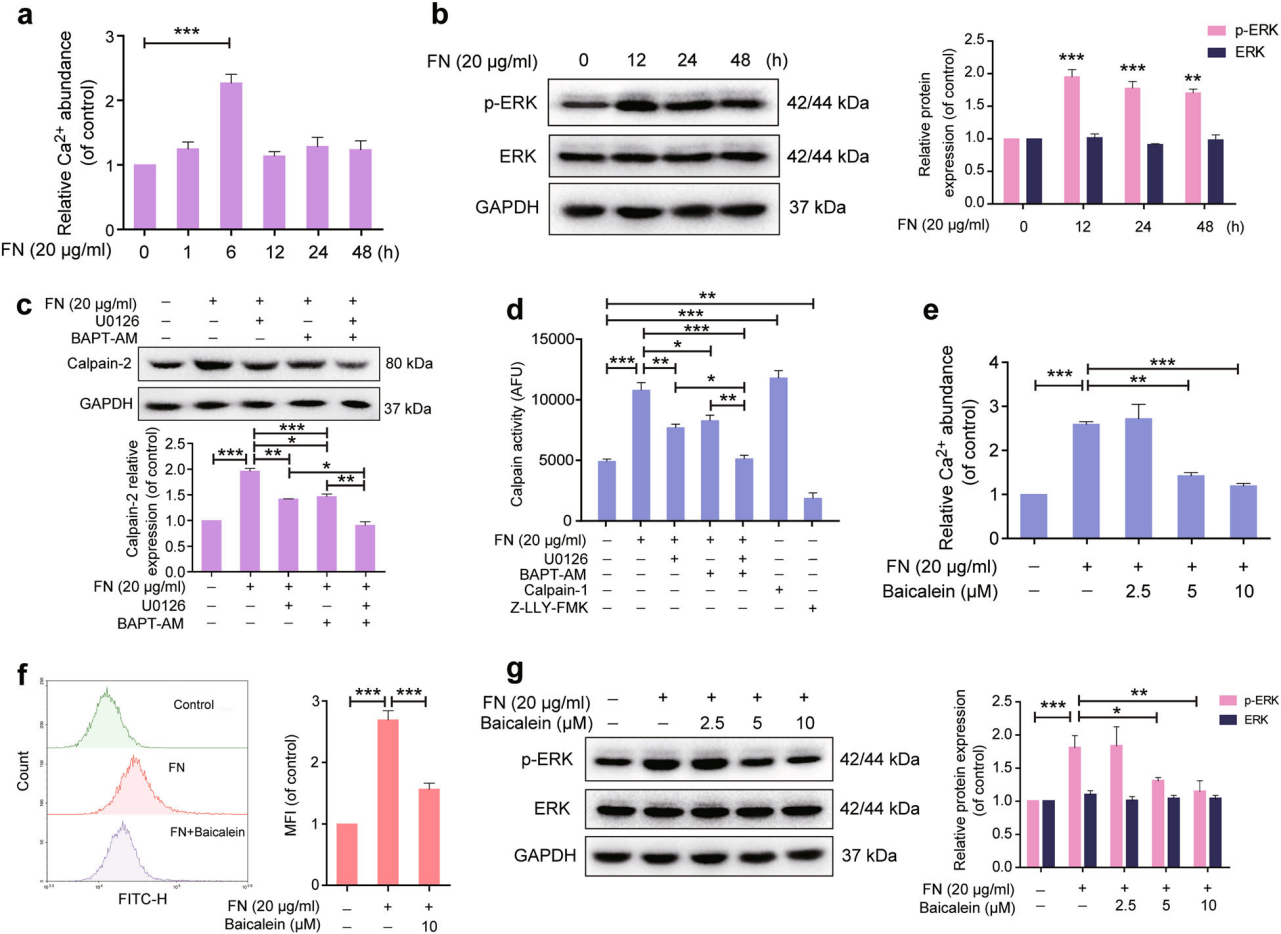
Effects of baicalein on fibronectin (FN)-induced intracellular elevation of Ca^2+^ and ERK activation. **a** MCF-10A cells were exposed to FN (20 μg ml^−1^) for 0–48 h. Ca^2+^ levels were detected using a Ca^2+^ quantification kit with a fluorescence microplate reader at Ex/Em = 540/590 nm. **b** ERK1/2 phosphorylation increased in MCF-10A cells after treatment with FN (20 μg ml^−1^) for 0–24 h. GAPDH was used as loading control. **c** MCF-10A cells were pretreated with U0126 (10 µM) and BAPT-AM (10 µM) for 30 min and then plated on FN (20 μg ml^−1^) for 48 h. Calpain-2 expression was detected by western blotting. GAPDH was used as loading control. **d** Calpain activity was expressed as arbitrary fluorescence units (AFUs) and measured by fluorescence assay (Ex/Em = 400/505 nm). **e** MCF-10A cells were exposed to FN (20 μg ml^−1^) in the presence or absence of baicalein at the indicated concentrations for 6 h, and Ca^2+^ levels were then detected using a Ca^2+^ quantification kit. **f** Cytosolic Ca^2+^ levels were measured with Fluo-3 AM and treated with FN in the presence or absence of 10 μM baicalein for 6 h by flow cytometry. Histogram overlays displaying fluorescence intensities (left) and mean fluorescence intensity (MFI, right) after treatment are shown. **g** Baicalein suppressed FN-induced ERK1/2 phosphorylation. Cells were treated with FN (20 μg ml^−1^) with or without baicalein at the indicated concentrations for 12 h. GAPDH was used as loading control. The results are shown as fold-change compared with control cells. Data are shown as mean ± SEM for three separate experiments. *P < 0.05, **P < 0.01, ***P < 0.001

Ca^2+^ binding to calpain results in a conformational switch to form a functional catalytic site^19^. Furthermore, the upstream effector ERK has been shown to mediate calpain-2 activation induced by EGF, which may occur in the absence of cytosolic Ca^2+^ fluxes^32^. We further explored how baicalein retarded FN-enhanced calpain activity by examining its effects on intracellular Ca^2+^ abundance and ERK phosphorylation in MCF-10A cells. Ca^2+^ abundance increased remarkably after exposure to FN for 6 h (Fig. 3a), and ERK phosphorylation was increased after FN treatment from 12 to 48 h, most obviously at 12 h (Fig. 3b). In addition, the highly selective MEK1/2 inhibitor U0126 and the intracellular Ca^2+^ chelator BAPT-AM had only partial effects on reducing FN-enhanced calpain-2 expression and activation, whereas the two agents combined completely abolished this effect (Fig. 3c, d), indicating that FN induced calpain-2 expression and activation via both ERK activation and Ca^2+^ increase. However, the intracellular Ca^2+^ increase was inhibited by baicalein (Fig. 3e), as confirmed by the Ca^2+^-sensitive fluorescence indicator Fluo-3 AM and flow cytometry (Fig. 3f). Baicalein also significantly impaired the FN-induced effect on ERK phosphorylation (Fig. 3g). In addition, baicalein reduced EGF (ERK stimulation) and ionomycin (Ca^2+^ stimulation) induced calpain-2 activity and expression (Supplementary Fig. S3c and d).

### Baicalein suppresses FN-induced EMT in a calpain-2-dependent manner

**Fig. 4:**
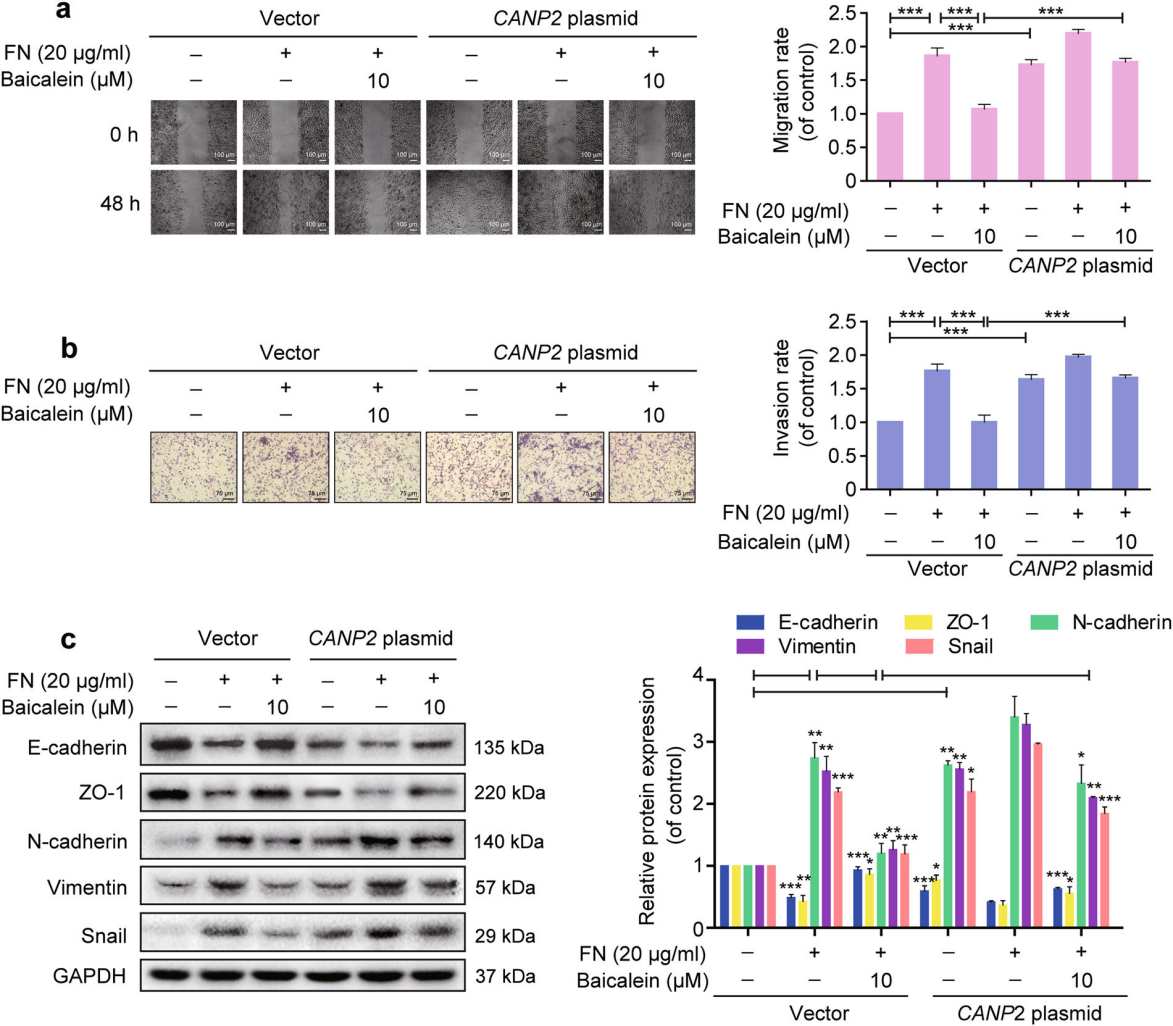
Baicalein suppresses fibronectin (FN)-induced epithelial–mesenchymal transition (EMT) in a calpain-2-dependent manner. Overexpression of calpain-2 reversed the inhibitory effects of baicalein on FN-induced EMT response in MCF-10A cells. **a** Cell migration ability was measured using a wound healing assay (magnification, ×100, scale bars: 100 μm). **b** Cell invasion was assessed using the Matrigel-coated transwell model (magnification, ×200, scale bars: 75 μm). **c** EMT markers were detected by western blotting and normalized to GAPDH expression. The results are shown as shown as fold-change compared with control cells. Data are shown as mean ± SEM from three separate experiments. *P < 0.05, **P < 0.01, ***P < 0.001

The role of calpain-2 in baicalein suppression of the FN-induced EMT response was further confirmed by transfecting MCF-10A cells with a plasmid expressing the calpain-2 gene (*CAPN2*) to generate calpain-2-overexpressing cells. As expected, calpain-2 expression was significantly increased after transfection, and this increase was not prevented by baicalein (Supplementary Fig. S4). Overexpression of calpain-2 significantly increased cell migration and invasion, and prevented the inhibitory effects of baicalein on FN-enhanced cell migration and invasion (Fig. 4a, b). The inhibitory effects of baicalein on FN-induced E-cadherin and ZO-1 downregulation and N-cadherin, vimentin, and Snail upregulation were also reduced in calpain-2-overexpressing cells (Fig. 4c). Overall, these results indicate that baicalein inhibited the FN-induced EMT response via calpain-2 suppression.

### Baicalein inhibits breast tumor onset, progression, and lung metastases and prolongs survival in MMTV-PyMT mice

**Fig. 5:**
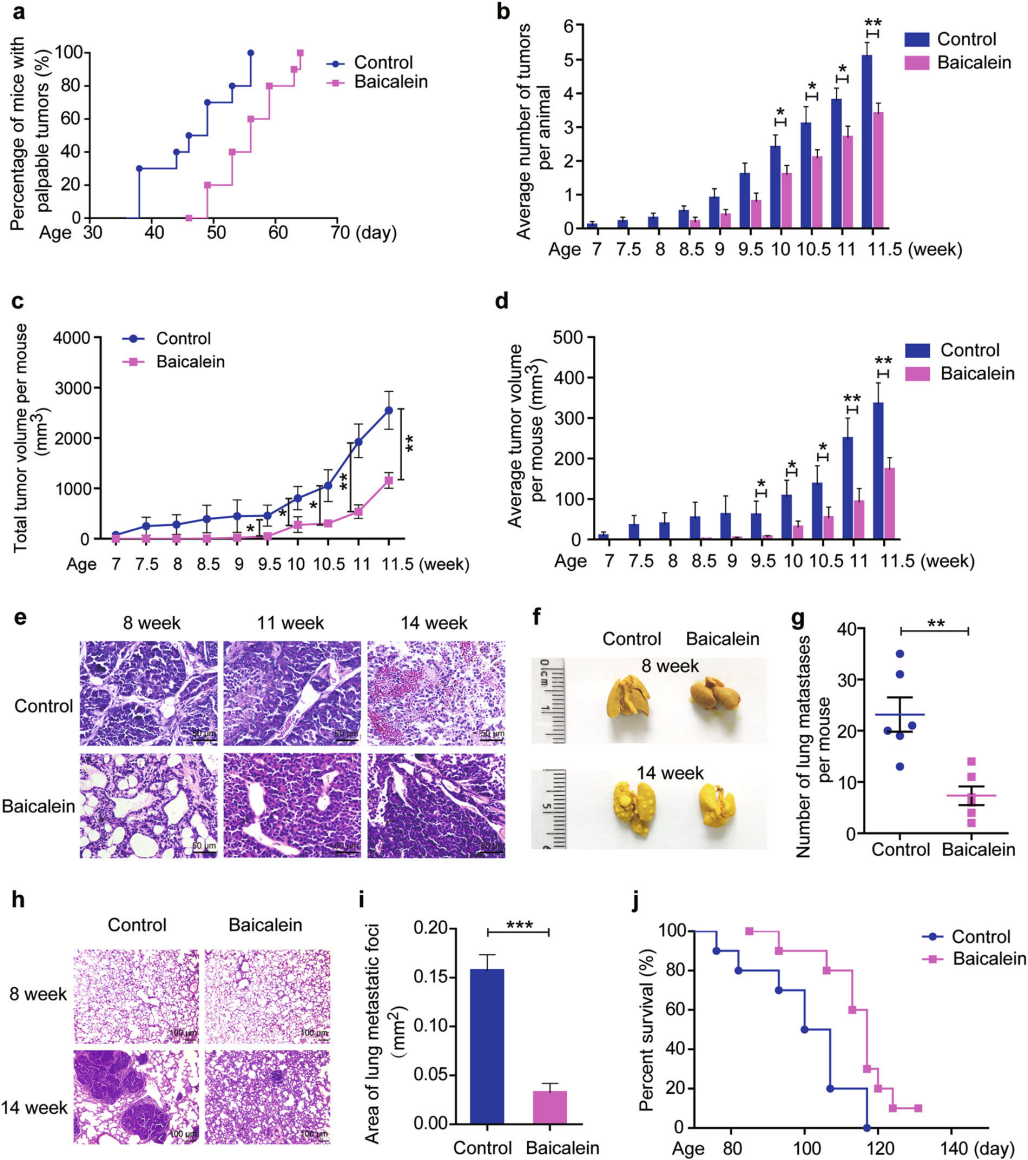
Effects of baicalein on tumor onset, growth, and lung metastasis in MMTV/PyMT mice. Female MMTV-PyMT transgenic mice were treated with vehicle or baicalein (30 mg kg^−1^) intraperitoneally after weaning (at the fourth week) twice weekly (*n* = 10 per group). Tumor number and volume were measured twice a week. **a** Kaplan–Meier survival plot for the percent of mice with palpable tumors. The curves differed significantly between the baicalein-treated and the control groups (*n* = 10, log-rank test). **b** Average number of tumors per animal (*n* = 10). **c** Total tumor volume per animal (*n* = 10). **d** Average tumor volume per animal (*n* = 10). **e** Representative images of tumor sections stained with H&E, ×400. Scale bars: 50 μm. **f** Representative photographs of dissected lungs (×1). **g** Number of lung metastatic nodules per mouse (*n* = 6). **h** H&E staining of metastatic lung tissues (×100). Scale bars: 100 μm. **i** Area of lung metastatic foci (*n* = 6). Results represent the mean areas of lung metastatic foci from photographs of H&E-stained sections (*n* = 6). **j** Kaplan–Meier survival plot for MMTV-PyMT mice. The survival curves differed significantly between the baicalein-treated and control groups (*n* = 10, log-rank test). Data are shown as mean ± SEM. *P < 0.05, **P < 0.01, ***P < 0.001

We determined if baicalein could mitigate breast cancer development in MMTV-PyMT transgenic mice, as a spontaneous model of breast cancer. All 10 mammary glands of each mouse were palpated twice weekly to evaluate the effects of baicalein on tumor onset and growth. Palpable tumors beneath the nipple were first detected at 38 days old in the control group, but not until 46 days old in the baicalein-treated group (Fig. 5a). All mice were tumor bearing at 56 and 64 days old in the control and baicalein-treated groups, respectively. Kaplan–Meier analysis for the appearance of tumors showed that baicalein significantly delayed the spontaneous onset of mammary gland tumors (log-rank test, *P* = 0.0041, < 0.01). The number of tumors per mouse was lower in baicalein-treated MMTV-PyMT compared with control mice (Fig. 5b). Furthermore, baicalein-treated mice displayed significantly smaller total and average tumor volumes per mouse compared with the controls (Fig. 5c, d). Hematoxylin and eosin (H&E) staining of tumor tissues from MMTV-PyMT mice showed advanced cellular proliferation and packed acini and ducts at 8 weeks old, neovascularization at 11 weeks, and more significant neovascularization and marked variation of cell morphology at 14 weeks (Fig. 5e). However, these tumorigenic characteristics were reversed by baicalein administration (Fig. 5e). Moreover, treatment with baicalein 30 mg kg^−1^ twice a week for 7 weeks had no obvious toxic effects on the main organs in MMTV-PyMT mice (Supplementary Fig. S5 and Table S1).

MMTV-PyMT mice treated with baicalein presented with significantly fewer lung metastatic nodules than control mice at 14 weeks old (7.3 ± 1.8 compared with 23.2 ± 3.4, respectively) (Fig. 5f, g). The average area of lung metastatic foci was significantly decreased at 14 weeks of age after administration of baicalein (0.033 ± 0.009 mm^2^ compared with 0.16 ± 0.02 mm^2^), as confirmed by H&E staining (Fig. 5h, i). Considering that approximately 90% of cancer patient deaths are caused by metastasis^33^, we explored the effect of baicalein on survival in this mouse model. Baicalein treatment significantly prolonged the median survival duration of the mice (117 vs. 103.5 days, log-rank test, *P* = 0.0153 < 0.05) (Fig. 5j).

### Baicalein treatment reverses increases in FN, calpain-2, and vimentin, and loss of E-cadherin in MMTV-PyMT mice

**Fig. 6:**
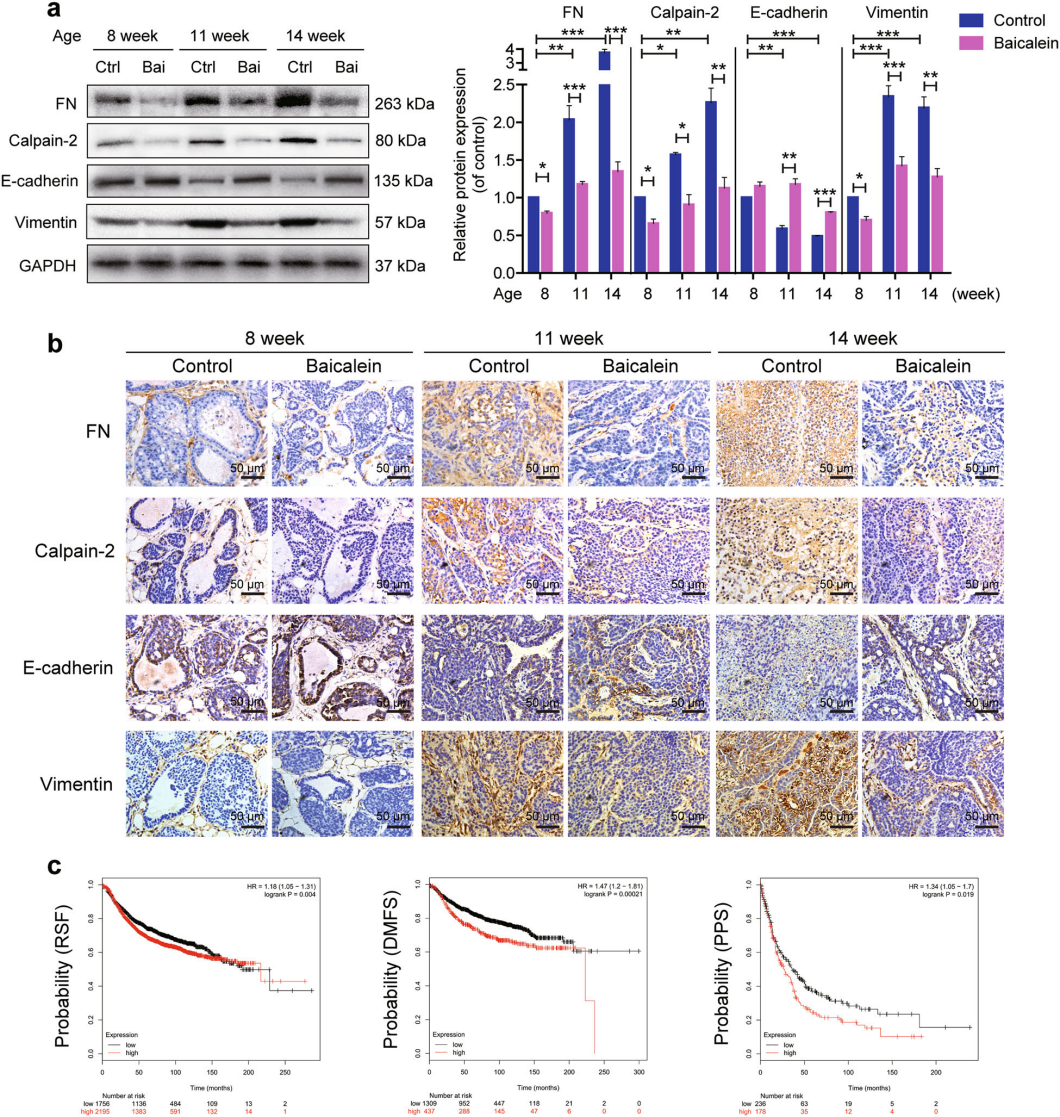
Effects of baicalein on fibronectin (FN), calpain-2, E-cadherin, and vimentin expression in breast tissues of MMTV-PyMT mice. **a** Protein extracts from tumors were analyzed by western blotting with FN, calpain-2, E-cadherin, and vimentin antibodies. Protein levels were quantified and normalized to GAPDH. The results are shown as fold-change compared with control (Ctrl) cells. Data are presented as mean ± SEM for three separate experiments. *P < 0.05, **P < 0.01, ***P < 0.001. **b** Representative images of tumor sections immunostained with anti-mouse-FN, anti-mouse-calpain-2, anti-mouse-E-cadherin, or anti-mouse-vimentin antibodies (×400). Scale bars: 50 μm. **c** FN expression correlated with survival of breast cancer patients. Kaplan–Meier survival curves suggested that high FN expression was associated with poor relapse-free survival (RFS, *n* = 3951), distant metastasis-free survival (DMFS, *n* = 1746), and palliative performance scale (PPS, *n* = 414) compared with lower FN expression

We determined if the effects of baicalein on the expression of FN, calpain-2, E-cadherin, and vimentin in breast tumor tissue reflected the in vitro results. Protein levels in tumor sections were examined at various time points during tumor progression in 8- to 14-week-old MMTV-PyMT mice. FN expression was notably increased from early- (8 weeks) to late-stage tumor development (14 weeks) in the control group, as indicated by western blot (Fig. 6a). Consistent with the western blot results, immunohistochemistry revealed that FN-positive clusters increased gradually with tumor progression, with especially high intensity in tumors in the control group at 14 weeks (Fig. 6b). We also examined the relationship between FN gene expression and clinical outcomes in breast cancer patients using the Kaplan–Meier Plot database. The results showed that high expression of FN was significantly correlated with poor relapse-free survival (RFS) (*n* = 3951), distant metastasis-free survival (DMFS) (*n* = 1746), and palliative performance score (PPS) (*n* = 417) in patients (Fig. 6c). In contrast, FN expression significantly decreased in breast tissue from baicalein-treated compared with control mice at the same interval (Fig. 6a, b). Calpain-2 expression was increased in breast tumors in accordance with the increase in FN expression. The elevated calpain-2 expression was mitigated by baicalein (Fig. 6a, b). Notably, EMT markers changed during cancer progression, specifically with loss of E-cadherin and increase of vimentin, but these changes were significantly attenuated by baicalein (Fig. 6a, b).

## Discussion

The important role of EMT in promoting cancer progression and metastasis indicates that pharmacological interventions targeting this process may provide new therapeutic strategies for breast cancer^34^. EMT of breast cancer cells can be triggered by a cluster of signals from the tumor microenvironment^35^. FN is a critical multifunctional glycoprotein of the ECM in the tumor microenvironment, and acts as a potent mediator in tumor proliferation, differentiation, morphogenesis, cell–matrix and cell–cell adhesion, migration, and oncogenic transformation^5,8^. In addition, FN stimulates an EMT response in mammary epithelial and breast cancer cells^17,25^. In accordance with previous results, the present study showed that FN, but not other tested ECM protein substrates including collagen IV and laminin, induced decreases in E-cadherin and increases in vimentin. In addition to its effects on EMT markers, FN also enhanced cell migration and invasion behaviors and mesenchymal phenotype changes via rearrangement of the F-actin network. The tumor progression stages in MMTV/PyMT mice have been well defined, from hyperplasia, mammary intraepithelial neoplasia, to non-invasive adenoma and finally invasive carcinoma^36^. The current in vivo results showed that breast cancer progression was accompanied by increased FN and EMT changes in MMTV-PyMT mice, with significant increases in FN expression in tumor tissues from 8 to 11 weeks old, reflecting the results in human breast tumor tissue^37^. E-cadherin and vimentin were also down and upregulated, respectively, in late-stage compared with early-stage tissues. These results indicate that FN-induced EMT might be a critical process in breast cancer progression.

The potential of flavonoids in breast cancer chemoprevention and therapy has attracted much attention^38^. Baicalein, a natural flavonoid from the Chinese medicinal plant Huang Qin, has been identified by preclinical studies as a novel and promising anti-breast cancer agent^26^. We demonstrated that baicalein inhibited FN-induced EMT changes in MCF-10A cells; specifically, baicalein depressed FN-enhanced migration, invasion, and F-actin rearrangement, and suppressed FN-induced downregulation of the epithelial markers E-cadherin and ZO-1, and FN-induced upregulation of the mesenchymal markers N-cadherin, vimentin, and Snail. The addition of FN to three-dimensional cultures stimulated polarized and growth-arrested mammary acini to proliferate and form disordered structures, suggesting that FN contributes to breast tumorigenesis^10^. FN promotes breast cancer invasion by enhancing matrix metalloproteinase 9 secretion^39^. Furthermore, FN expression is significantly correlated with an invasive and metastatic phenotype and serves as a prognostic biomarker for breast cancer^7,8,26^. Kaplan–Meier analysis of a public database confirmed that patients with higher expression of FN had poorer clinical outcomes in terms of RFS, DMFS, and PPS compared with patients with lower expression. Fibrin–FN complexes have been demonstrated to support the retention of tumor cells in the lungs and promote tumor cell invasion, eventually facilitating lung metastasis^40^. These investigations suggest that increased FN levels may cause both breast tumor initiation and progression. The present results revealed that baicalein decreased the incidence and size of pulmonary metastasis and prolonged survival in MMTV/PyMT mice after treatment at the early stage. Consistent with the in vitro results, baicalein reduced FN and vimentin expression but increased E-cadherin expression in breast tumor tissue of MMTV-PyMT mice compared with control mice at the same stage. These results indicate that baicalein impaired tumor initiation and progression though suppressing the function of FN, potentially related to the inhibition of FN-induced EMT. FN directly stimulates mammary epithelial cell proliferation^10^ or modulates vascular endothelial growth factor-mediated signaling and blood vessel formation, and subsequently promotes tumor growth^41^. Baicalein significantly decreased tumor growth in MMTV/PyMT mice in the current study. However, cell proliferation was unaffected by the concentrations of baicalein and FN used in our in vitro experiments. Baicalein exerts its antitumor effects via complicated mechanisms, and its role and mechanisms in relation to FN-induced cell growth require further investigation.

Calpain activity is required for limited proteolysis of several substrates, such as paxillin, FAK, talin, cortactin, and spectrin, which facilitates cytoskeletal remodeling, focal adhesion disassembly, membrane protrusions, and invadopodia/podosome formation^42–44^. Enhanced calpain expression and activity augment cell motility and invasion in several types of cancer^20^. Calpain is also a downstream factor for FN signaling. It transduces FN-induced FAK signaling activation in lung cancer cell migration and invasion^24^, and mediates FN-induced spreading and formation of focal adhesions, actin filament networks, and stress fibers in bovine aortic endothelial cells^45^. Furthermore, the calpain inhibitors calpeptin, MG-101 (ALLN), and calpain inhibitor IV suppressed FN-promoted migration, invasion, and EMT marker changes in breast cancer cells^25^. Inhibition of calpain might thus provide a potential strategy for suppressing FN-induced EMT changes. In the present study, calpain-2, but not calpain-1, was significantly upregulated in response to FN stimulation. Calpain-2 expression increased gradually with breast cancer progression in MMTV-PyMT mice, in accordance with expression changes in FN and EMT markers. Moreover, FN promoted calpain activity, indicated by increased cleavage of its fluorescence substrate Ac-LLY-AFC, membrane localization, and the generation of low-molecular-weight calpastatin. Baicalein treatment significantly reduced FN-induced upregulation of calpain-2 expression and activity in MCF-10A cells; however, the inhibitory effects of baicalein on FN-enhanced migration, invasion, and EMT markers were abolished in cells transfected with a calpain-2-overexpression plasmid. Furthermore, baicalein also decreased the expression of calpain-2 in breast tumors in MMTV-PyMT mice. These results suggested that baicalein suppressed FN-induced EMT by inhibiting calpain-2 expression and activation.

The results of this study indicate that FN-enhanced calpain-2 expression and activation may require both Ca^2+^ increase and ERK activation. Baicalein suppressed FN-induced ERK phosphorylation and cellular Ca^2+^ increases, indicating that it may depress FN-enhanced calpain-2 expression and activation through inhibition of these two pathways. However, further investigations are required to identify the detailed molecular mechanisms of baicalein-induced calpain-2 inhibition. Furthermore, α5β1 integrin is the FN receptor and transmits FN signals from the ECM^46^, and calpain-2 acts as a downstream signaling molecule of α5β1 integrin^47^; however, the role of α5β1 integrin in FN-induced activation and upregulation of calpain-2, and the effects of baicalein on FN–integrin α5β1 signaling remain to be elucidated.

Although many investigations have confirmed the anti-breast cancer activities of baicalein in vitro and in vivo, the present study provides the first preclinical evidence for the therapeutic potential of baicalein in MMTV-PyMT mice a spontaneous model of breast cancer, closely mimicking the progression of the human disease^36,48^. Several preclinical and clinical studies have revealed that baicalein has a good safety profile with no signs of mutagenesis or genomic instability^49^. A single oral dose of baicalein of 100–2800 mg was shown to be safe and well-tolerated with no serious adverse events in a randomized, single-dose, double-blind phase I clinical trial^50^. We showed that baicalein caused no obvious toxicity in the main organs, such as the heart, liver, spleen, and kidney, in MMTV-PyMT mice after continuous treatment with 30 mg kg^−1^ twice a week for 7 weeks. However, the long-term safety and toxicological profiles of baicalein require further investigation to determine its suitability for the clinical treatment of breast cancer.

**Fig. 7:**
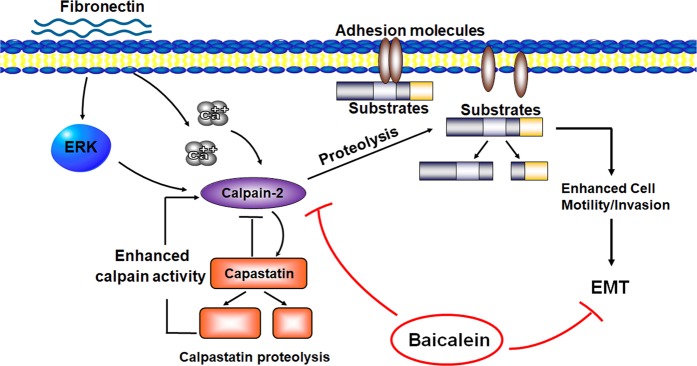
**A schematic diagram depicting the possible mechanism underlying the inhibitory effects of baicalein on fibronectin (FN)-induced epithelial–mesenchymal transition (EMT) in breast cancers**

The results of the current investigation confirmed the potential of baicalein as an inhibitor of breast cancer onset, progression, and metastasis in a clinically relevant transgenic mouse model. Baicalein significantly suppressed FN-induced EMT and calpain-2 activation and upregulation (Fig. 7). Targeting EMT is becoming an increasingly common approach in tumor therapy, and treatment with baicalein to reduce FN-induced EMT could thus represent a new strategy for overcoming breast cancer initiation and progression.

## Materials and methods

### Reagents

Baicalein (456119), FN (F2006), collagen IV (C5533), and laminin (L6274) were obtained from Sigma-Aldrich (St. Louis, MO, USA). Baicalein was dissolved in dimethyl sulfoxide to produce a stock solution (0.1 M) and stored at −20 °C. Matrigel (356237) was obtained from BD Biosciences (Bedford, MA, USA). The membrane and cytosol protein extraction kit was purchased from Beyotime Biotechnology (P0033; Jiangsu, China). Primary antibodies against E-cadherin (R868) (BS1098, 1:1000), vimentin (I444) (BS1491, 1:1000), N-cadherin (W745) (BS2224, 1:1000), ERK1/2 (L352) (BS1112, 1:1000), phosphorylated-ERK (T202/Y204) (AP0484, 1:1000), Na^+^/K^+^-ATPase ɑ1 (Y10) (BS1436, 1:1000), and GAPDH (1A6) (MB001, 1:1000) were purchased from Bio-World (Dublin, OH, USA). Primary antibodies against ZO-1 (#8193, 1:1000) and Snail (#3879, 1:1000) were purchased from Cell Signaling Technology (Beverly, MA, USA). Primary antibodies for calpain-1 (H-65) (sc-13990, 1:1000), calpain-2 (E-10) (sc-373966, 1:1000), and calpastatin (H-300) (sc-20779, 1:1000) were purchased from Santa Cruz Biotechnology, Inc. (Santa Cruz, CA, USA). Primary anti-mouse-FN (M168) (ab45688, 1:10000), anti-mouse-E-cadherin (ab76055, 1:1000), anti-mouse-vimentin (ab92547, 1:5000), and anti-mouse-calpain-2 (ab39165, 1:5000) used in the animal experiments were purchased from Abcam, Inc. (Cambridge, MA, USA). Fluorescein isothiocyanate (FITC)-labeled goat anti-rabbit secondary antibody (BS10950, 1:100) was purchased from Bio-World. Horseradish peroxidase (HRP)-conjugated anti-mouse (sc-2302) and anti-rabbit (sc-2030) IgG secondary antibodies (1:5000) were purchased from Santa Cruz Biotechnology, Inc.

### Cell culture and treatment

The MCF-10A cell line was purchased from the American Type Culture Collection (ATCC, Manassas, VA, USA). MCF-10A cells were incubated in culture medium with 5% horse serum (1312396, Gibco, Carlsbad, CA, USA). The culture medium comprised a 1:1 mixture of Ham’s F12 medium and Dulbecco’s modified Eagle’s medium (11330032, Gibco), insulin (10 μg ml^−1^, I6634, Sigma), EGF (20 ng ml^−1^, AF-100-15, PeproTech, London, United Kingdom), hydrocortisone (0.5 μg ml^−1^, H0888, Sigma), and cholera enterotoxin (0.1 μg ml^−1^, C8052, Sigma). The cells were incubated at 5% CO_2_ at 37 °C. Treatment with FN, Matrigel, collagen IV, or laminin was carried out in culture medium supplemented with 1% horse serum. The cells were starved in culture medium with 1% serum for 12 h and then allowed to spread on FN, Matrigel, collagen IV, or laminin pre-coated coverslips or culture plates with different concentrations of baicalein for 0–48 h.

### Wound-healing assays

The cells were spread on FN pre-coated plates to form a monolayer, and the monolayer was then wounded using a micropipette tip. Photographs were taken at 0 and 48 h after wounding using a Leica DMI 1 microscope and LAS EZ software (Leica, Wetzlar, Germany) at ×100 magnification.

### Invasion assay

Cell invasion ability was estimated using a Matrigel-coated transwell chamber with an 8-μm pore size membrane (R3NA43983, Millipore, Billerica, MA, USA) as described previously^51^. After trypsinization, the cells were suspended in serum-free medium (5 × 10^5^ ml^−1^) and added into the upper chamber, and medium containing 5% horse serum was added into the lower chamber. After 24-h incubation, the non-invaded cells on the upper surface of the membrane were removed. Cells that had invaded through the Matrigel onto the lower surface were fixed in 100% methanol and stained with H&E. Images were captured using a Leica DMI 1 microscope and LAS EZ software (Leica) at ×200 magnification.

### Western blotting

After treatment, the cells were lysed in lysis buffer (Beyotime), and the lysates were centrifuged at 13,000 × *g* for 15 min at 4 °C. The protein concentration in the supernatants was detected using a bicinchoninic acid assay kit (Pierce, Rockford, IL, USA) with a microplate spectrophotometer (Varioskan LUX, Thermo Fisher Scientific, Vantaa, Finland). Total proteins (30 μg per lane) were separated using 12% sodium dodecyl sulfate-polyacrylamide gel electrophoresis, and transferred to a polyvinylidene fluoride membrane (Millipore). The membrane was blocked with 5% non-fat milk and subsequently incubated with the appropriate antibodies, as described in Reagents above. The results were captured using a ChemiDoc XRS^+^ system and analyzed with Image Lab™ Software version 5.2 (Bio-Rad, Hercules, CA, USA).

### Immunofluorescence microscopy

Coverslips were fixed with 4% paraformaldehyde for 15 min and permeabilized with 0.2% Triton X-100 for 15 min followed by blocking with 10% goat serum (Dako, Carpinteria, CA, USA) for 1 h. The cells were then stained with Phalloidin-iFluor 488 (ab176753, Abcam) for 1 h to analyze F-actin remodeling. For E-cadherin, N-cadherin, and vimentin analysis, the coverslips were incubated with primary antibodies (1:50) at 4 °C overnight, followed by FITC-conjugated secondary antibodies for 1 h and 4, 6′-diamidino-2-phenylindole dihydrochloride (DAPI, 1 μg ml^−1^, Bio-World) for 20 min. E-cadherin and N-cadherin images were captured using a Leica DMI8 microscope and Leica X software (Leica) at ×200 magnification, and F-actin and vimentin images were photographed using a TCS SP5 confocal microscope and LAS AF software version Beta (Leica) at ×800 magnification.

### Measurement of calpain activity

Calpain activity was measured using a calpain activity assay kit (ab65308, Abcam) according to the standard manufacturer’s protocol. The calpain substrate Ac-LLY-AFC emits blue light (λmax = 400 nm) and releases free AFC that emits yellow-green fluorescence (λmax = 505 nm) upon cleavage by calpain. AFC release was measured using a microplate spectrophotometer at Ex/Em = 400/505 nm (Varioskan LUX, Thermo).

### Measurement of intracellular Ca^2+^ level

Intracellular Ca^2+^ levels were quantified using a Ca^2+^ quantification kit (Abcam, ab112115) following the standard manufacturer’s protocol. Fluorescence signals were detected using a microplate spectrophotometer at Ex/Em = 540/590 nm (Varioskan LUX, Thermo). Cytosolic Ca^2+^ levels were also measured by flow cytometric estimation of Fluo-3 AM. The cells were collected and loaded with 3 μM Fluo-3 AM (Abcam, ab145254) for 1 h at 37 °C in the dark, and then resuspended with 500 μl phosphate-buffered saline. The fluorescence signal was recorded using a flow cytometer at Ex/Em = 488/525 nm and analyzed by NovoExpress software (NovoCyte, ACEA Biosciences, San Diego, CA, USA).

### Transfection of *CAPN2* plasmid

The plasmid pEX-3 containing the *CAPN2* gene (NM_001748.4) was constructed by GenePharma, Inc. (Shanghai, China) and transfection was performed using GenJet™ Plus DNA In Vitro Transfection Reagent (Signagen, MD, USA, SL100499) in accordance with the manufacturer’s protocol.

### Animal experiments

Animal experiments were conducted in accordance with the Declaration of Helsinki and the Regulations for Care and Use of Laboratory Animals of the State Food and Drug Administration of China. The experimental protocols were approved by the Animal Ethics Committee of Guizhou Medical University. Female MMTV-PyMT transgenic mice (about 3 weeks old) were obtained from Nanjing Biomedical Research Institute of Nanjing University (Nanjing, Jiangsu, China) and raised with standard laboratory food and water under 12:12 h light/dark cycle conditions.

The mice were randomized into two groups and injected intraperitoneally with baicalein (30 mg kg^−1^; dissolved in olive oil) or an equal volume of olive oil twice a week from 4 to 11 weeks old. Tumors were measured in all 10 mammary glands of each mouse twice weekly using calipers, and tumor volume was calculated as 0.5 × width × length × length. After CO_2_ inhalation, animals were killed at the indicated times and tumors were dissected, weighed, fixed in 10% neutral buffered formalin, and embedded in paraffin for later H&E or immunohistochemical staining. Lungs were excised from 8- to 14-week-old mice, fixed in Botteon’s buffer, and the total number of surface micrometastases was counted and imaged using a digital color camera (D7200, Nikon, Tokyo, Japan) and a 3,3′-diaminobenzidine (DAB) kit (ZLI-9017, Zhongshan Golden Bridge Biotechnology, Beijing, China).

### Histological analysis

Morphology was examined in tissue sections (5 μm) stained with H&E. The expression of FN, calpain-2, E-cadherin, and vimentin was analyzed using a two-step Immunohistochemical Stain Detection System (PV-9000, Zhongshan Golden Bridge Biotechnology) according to the manufacturer’s instructions. Briefly, the sections were deparaffinized, and antigen retrieval was achieved by microwave heating in 0.01 M citrate buffer for 20 min. The sections were sequentially incubated with 3% H_2_O_2_, blocked with goat serum (Dako) for 30 min, and incubated with the following primary antibodies at 4 °C overnight: FN (1:300), E-cadherin (1:250), calpain-2 (1:250), and vimentin (1:350). The sections were successively incubated with biotinylated secondary antibodies for 20 min and streptavidin-HRP for 2 min, and then stained with DAB substrate and counterstained with hematoxylin. Results were captured using a Leica DMI8 microscope and Leica X software (Leica) at ×400 magnification.

### Kaplan–Meier plotter analysis

The clinical relevance of FN expression (probe: 211719_at) in relation to breast cancer patient survival was analyzed using a public database that integrates clinical data and gene expression by Kaplan–Meier Plotter, in auto select best cutoff mode (http://kmplot.com)^52^. RFS was analyzed in 3951 breast cancer samples, DMFS in 1746 breast cancer samples, and PPS in 417 breast cancer samples. Patient samples were divided into two groups on the basis of high and low FN gene expression levels. Log-rank test P-values were calculated and P < 0.05 was considered significant.

### Data and statistical analysis

Data are expressed as means ± standard error of the mean (SEM) from at least three independent experiments. Comparisons among multiple groups were analyzed using one-way analysis of variance followed by the Bonferroni post hoc test, and comparisons between two groups were analyzed using two-tailed Student’s *t*-tests. Tumor onset and survival in MMTV-PyMT mice were analyzed by Kaplan–Meier analysis followed by the log-rank test to compare differences. *P* < 0.05 was considered to indicate a significant difference.

## Supplementary information

Supplementary Information

Figure S1

Figure S2

Figure S3

Figure S4

Figure S5

Table S1
