# Supplementary material for: Baicalein inhibits fibronectin-induced epithelial–mesenchymal transition by decreasing activation and upregulation of calpain-2
Source: Cell Death Dis. 2019 Apr 18;10(5):341. doi: 10.1038/s41419-019-1572-7 (PMC6472504; doi:10.1038/s41419-019-1572-7)
Supplement: Supplementary file 1 — Supplementary Information [file 41419_2019_1572_MOESM1_ESM.docx]

**Figure S1** The effects of FN and baicalein on cell viability.

**Figure S2** The effects of ECM protein substrates on EMT-related makers expression.

**Figure S3** FN enhanced calpain-2 expression and activity.

**Figure S4** Confirmation of the overexpression of calpain-2.

**Figure S5** Effects of baicalein on main organs of MMTV-PyMT mice.

**Table S1** Effects of baicalein on weights of the main organs from MMTV-PyMT mice.

**Figure S1** The cell viability was detected by 3-(4, 5-dimethylthiazol-2-yl)-2, 5-diphenyltetrazolium bromide (MTT) assay. The cells were treated with FN in the presence or absence of baicalein (2.5, 5 and 10 μM) for 48 h. The results are shown as cell viability compared with control (as 100%). Values are mean ± SEM for three separate experiments.

**Figure S2** The expression of E-cadherin and vimentin in MCF-10A cells plated on different ECM protein substrates. The cells were plated on collagen IV (Col, 20 μg/ml), Matrigel (MG, 20 μg/ml), laminin (LN, 20 μg/ml) or FN (20 μg/ml) for 24 or 48 h. Western blot analysis is shown for the expression of E-cadherin and vimentin. The results obtained were normalized to GAPDH expression and are shown as the fold-change compared with control (Ctrl) cells. Data are shown as mean ± SEM for three separate experiments. ^**^*P <* 0.01, ^***^*P <* 0.001.

**Figure S3** FN enhanced calpain-2 expression and activity. The MCF-10A cells were exposed to FN (20 μg/ml) for 0–48 h. **a** The calpain-2 expression was detected by western blotting. The results obtained from experiments were normalized to GAPDH expression and are shown as the fold-change compared with control cells. **b** Calpain activity was expressed as arbitrary fluorescence units (AFU) and measured by a fluorescence assay (Ex/Em = 400/505 nm). The MCF-10A cells were pretreated with baicalein (10 µM), U0126 (10 µM) or BAPT-AM (10 µM) for 30 min and then stimulated with EGF (100 ng/ml) for 6 h or ionomycin (1 μM) for 12 h. U0126 and BAPT-AM were used as positive control for the inhibition of EGF or ionomycin, respectively. **c** Baicalein inhibited EGF or ionomycin enhanced calpain-2 expression. GAPDH was used as loading control. **d** Baicalein inhibited EGF or ionomycin promoted calpain activity. Data are shown as mean ± SEM for three separate experiments. ^*^*P* < 0.05, ^**^*P* < 0.01, ^***^*P* < 0.001.

**Figure S4** Confirmation of the overexpression of calpain-2 was detected by western blotting. Vector or *CANP2* plasmid was transfected into MCF-10A cells, and the cells were then plated on FN (20 μg/ml) in the presence or absence of baicalein (10 µM) for 48 h. The results obtained from experiments were normalized to GAPDH expression and are shown as the fold-change compared with control cells. Data are shown as mean ± SEM for three separate experiments. ^**^*P* < 0.01, ^***^*P* < 0.001.

**Figure S5** Effects of baicalein on main organs of MMTV-PyMT mice. **a** Representative photographs of dissected organs (heart, liver, spleen and kidney) from 5, 8 and 11-week (w) old MMTV-PyMT mice by color camera (×1, D7200, Nikon, Tokyo, Japan). **b** H&E stains of the dissected organ sections in MMTV-PyMT mice. The heart, liver, spleen and kidney were excised, weighed, fixed in 10% neutral buffered formalin and embedded in paraffin. The organ cross-sections (5 μm) were stained with H&E and scanned by a Leica DMI8 microscope and Leica X software (Leica, Wetzlar, Germany) under bright-field light at 200 × magnification. Scale bars: 75 μm.

**Table S1** Effects of baicalein on weights of the main organs from MMTV-PyMT mice at different time point. Data are expressed as mean ± SEM (n=5).
