# Supplementary figures and images for: Baicalein inhibits fibronectin-induced epithelial–mesenchymal transition by decreasing activation and upregulation of calpain-2
Source: Cell Death Dis. 2019 Apr 18;10(5):341. doi: 10.1038/s41419-019-1572-7 (PMC6472504; doi:10.1038/s41419-019-1572-7)

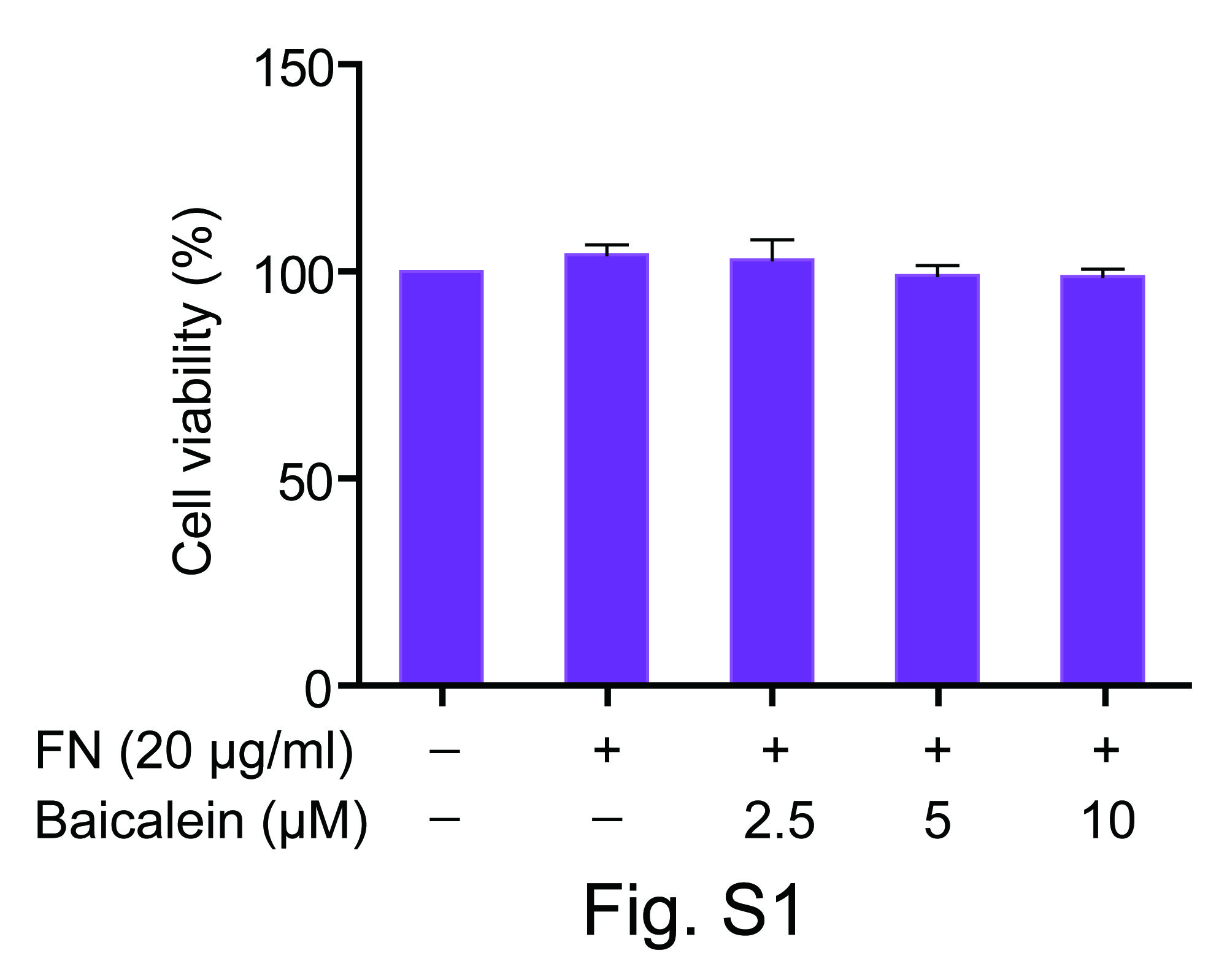

Supplement: Supplementary file 2 — Figure S1 [file 41419_2019_1572_MOESM2_ESM.jpg]

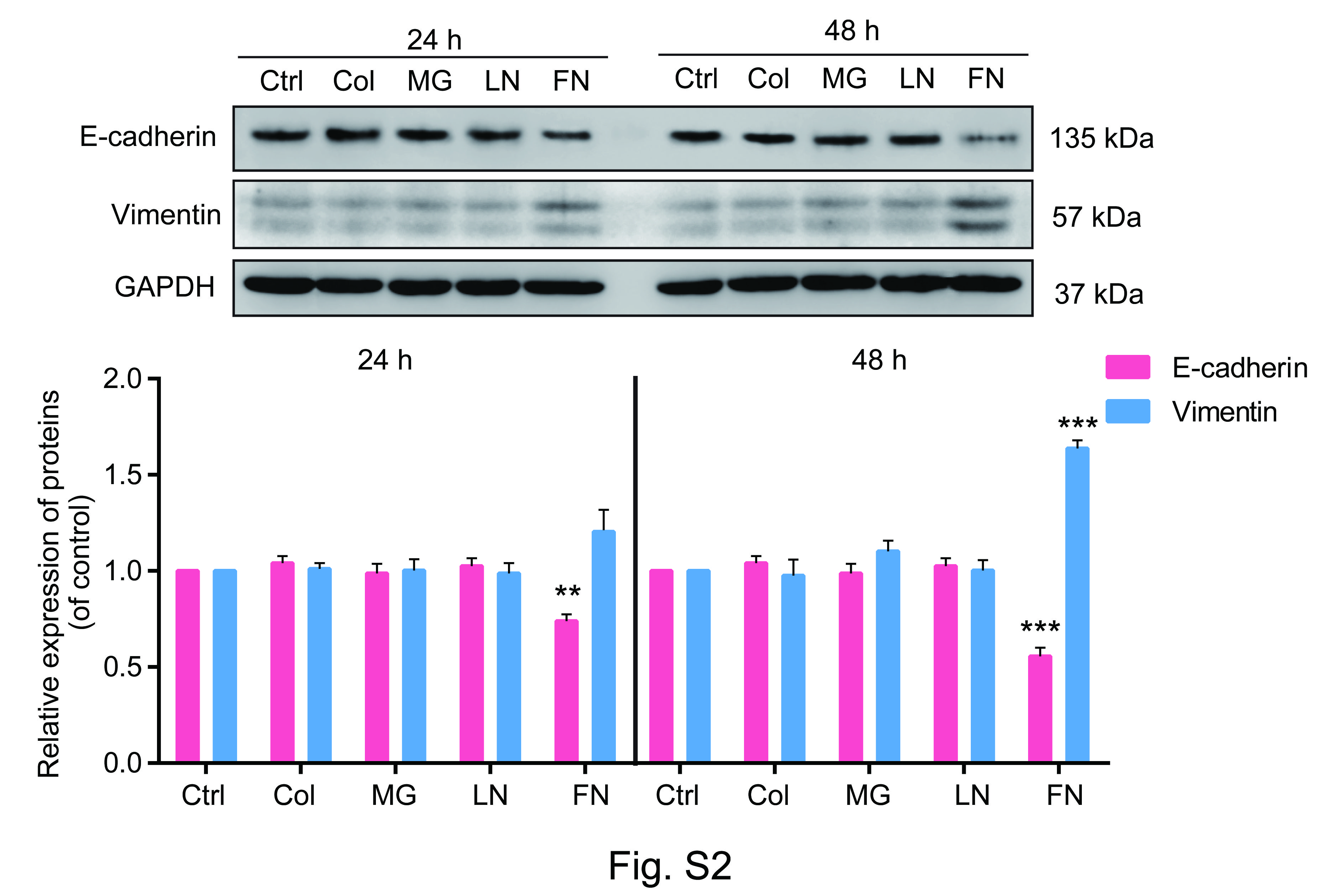

Supplement: Supplementary file 3 — Figure S2 [file 41419_2019_1572_MOESM3_ESM.jpg]

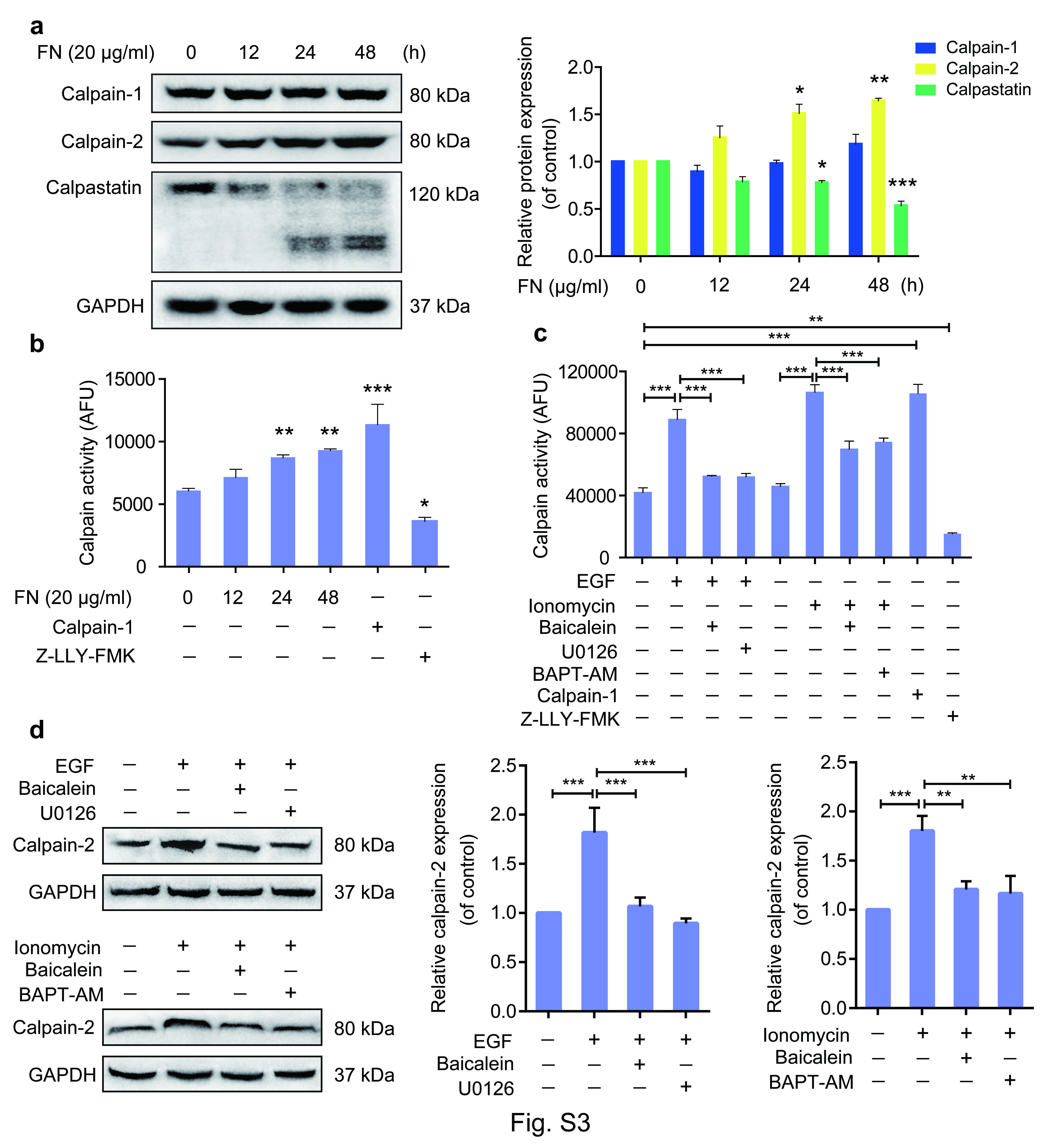

Supplement: Supplementary file 4 — Figure S3 [file 41419_2019_1572_MOESM4_ESM.jpg]

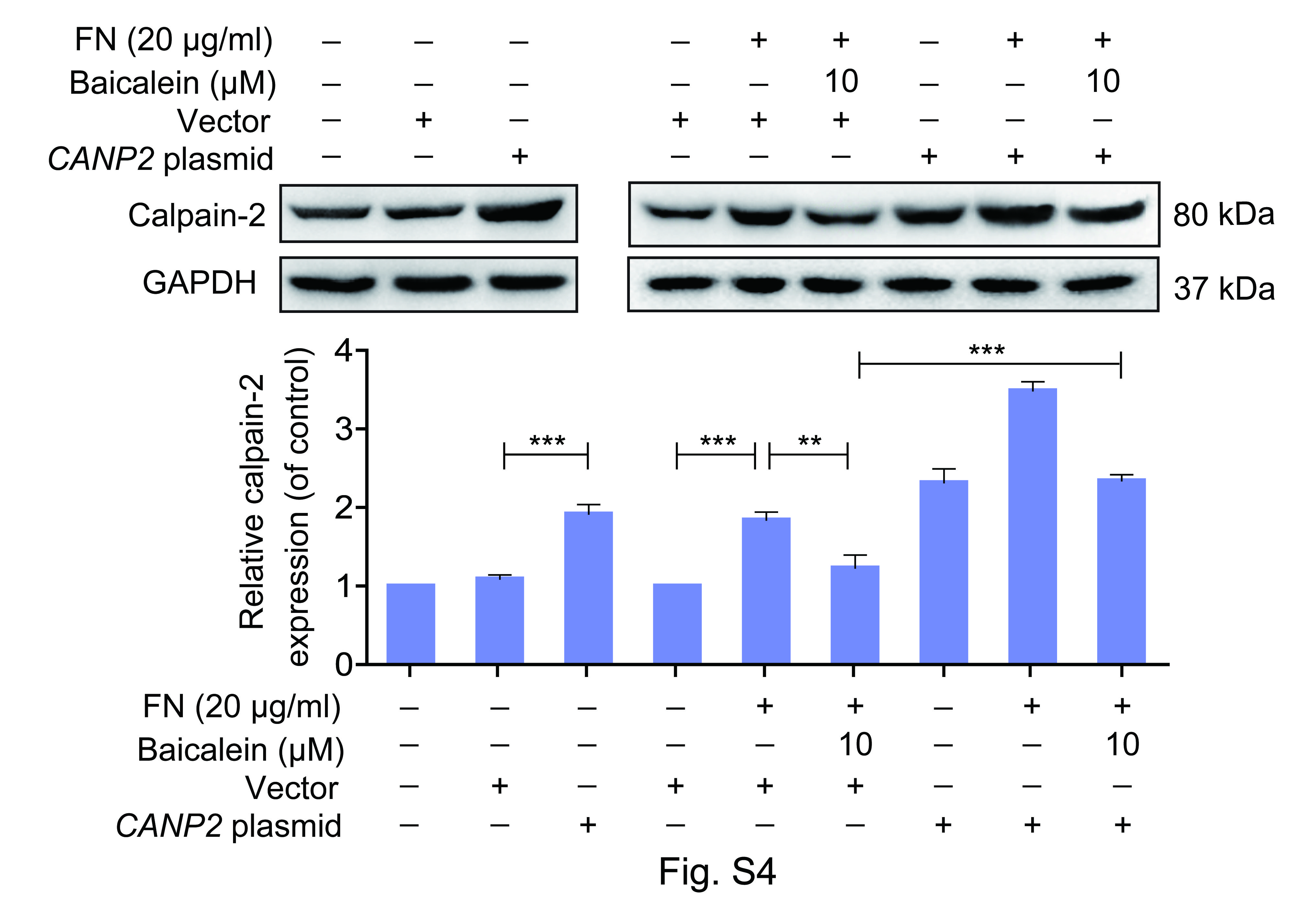

Supplement: Supplementary file 5 — Figure S4 [file 41419_2019_1572_MOESM5_ESM.jpg]

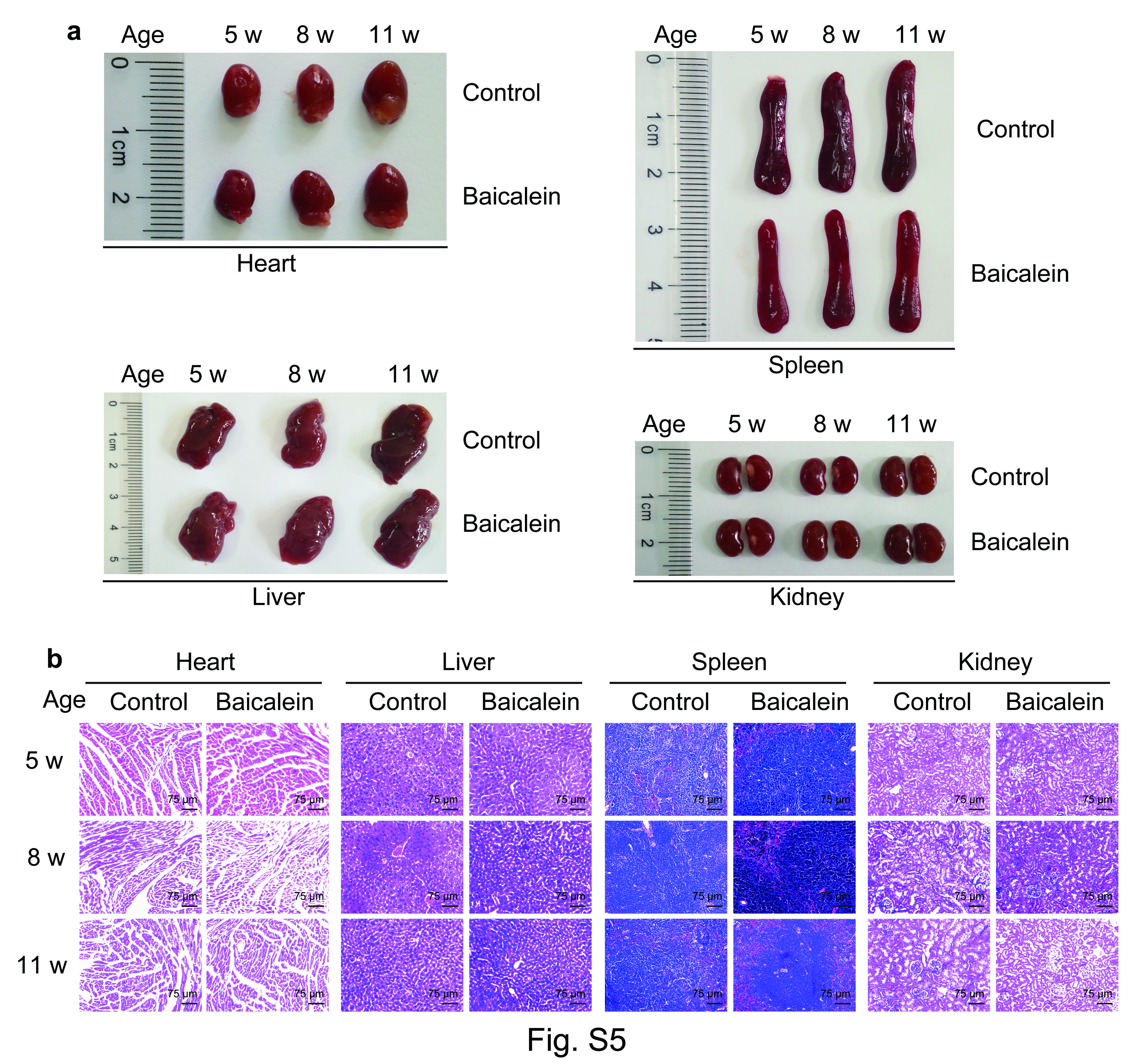

Supplement: Supplementary file 6 — Figure S5 [file 41419_2019_1572_MOESM6_ESM.jpg]
