# Supplementary material for: Baicalein inhibits fibronectin-induced epithelial–mesenchymal transition by decreasing activation and upregulation of calpain-2
Source: Cell Death Dis. 2019 Apr 18;10(5):341. doi: 10.1038/s41419-019-1572-7 (PMC6472504; doi:10.1038/s41419-019-1572-7)
Supplement: Supplementary file 7 — Table S1 [file 41419_2019_1572_MOESM7_ESM.docx]

­Supplementary Table S1 Effects of baicalein on weights of the main organs from MMTV-PyMT mice at different time point.

Data are expressed as mean ± SEM (n = 5).

| Age | Heart (g) | | | Liver (g) | | | Spleen (g) | | Kidney (g) | |
| --- | --- | --- | --- | --- | --- | --- | --- | --- | --- | --- |
|  | Control | Baicalein | Control | | Baicalein | Control | | Baicalein | Control | Baicalein |
| 5 weeks | 0.1216 ± 0.002 | 0.1255 ± 0.001 | 1.2266 ± 0.08 | | 1.3020 ± 0.09 | 0.1598 ± 0.02 | | 0.1615 ± 0.02 | 0.2396 ± 0.008 | 0.2590 ± 0.008 |
| 8 weeks | 0.1258 ± 0.008 | 0.1396 ± 0.01 | 1.3434 ± 0.07 | | 1.3330 ± 0.08 | 0.2338 ± 0.03 | | 0.2412 ± 0.02 | 0.2322 ± 0.01 | 0.2558 ± 0.03 |
| 11 weeks | 0.1886 ± 0.003 | 0.1827 ± 0.002 | 1.3212 ± 0.03 | | 1.2930 ± 0.02 | 0.2748 ± 0.02 | | 0.2560 ± 0.01 | 0.2430 ± 0.009 | 0.2353 ± 0.007 |
